# CHRR: coordinate hit-and-run with rounding for uniform sampling of constraint-based models

**DOI:** 10.1093/bioinformatics/btx052

**Published:** 2017-01-31

**Authors:** Hulda S Haraldsdóttir, Ben Cousins, Ines Thiele, Ronan M.T Fleming, Santosh Vempala

**Affiliations:** 1 Luxembourg Centre for Systems Biomedicine, University of Luxembourg, Belvaux, Luxembourg; 2 School of Computer Science, Algorithms and Randomness Center, Georgia Institute of Technology, Atlanta, GA, USA

## Abstract

**Summary:**

In constraint-based metabolic modelling, physical and biochemical constraints define a polyhedral convex set of feasible flux vectors. Uniform sampling of this set provides an unbiased characterization of the metabolic capabilities of a biochemical network. However, reliable uniform sampling of genome-scale biochemical networks is challenging due to their high dimensionality and inherent anisotropy. Here, we present an implementation of a new sampling algorithm, coordinate hit-and-run with rounding (CHRR). This algorithm is based on the provably efficient hit-and-run random walk and crucially uses a preprocessing step to round the anisotropic flux set. CHRR provably converges to a uniform stationary sampling distribution. We apply it to metabolic networks of increasing dimensionality. We show that it converges several times faster than a popular artificial centering hit-and-run algorithm, enabling reliable and tractable sampling of genome-scale biochemical networks.

**Availability and Implementation:**

https://github.com/opencobra/cobratoolbox.

**Supplementary information:**

Supplementary data are available at *Bioinformatics* online.

## 1 Introduction

A constraint-based model of a metabolic network, with *m* metabolites and *n* reactions, consists of a set of equalities and inequalities that define a set Ω of feasible steady state reaction rates, or fluxes, $v\in {\mathbb{R}}^{n}$. In the linear case,

(1) $$\Omega =\left\{v|Sv=0,l\leq v\leq u,c^{T}v=\alpha \right\}.$$

Here, $S\in {\mathbb{R}}^{m\times n}$ is a generalized incidence matrix known as a stoichiometric matrix. It is defined such that $S_{i,j}$ is the stoichiometric coefficient of metabolite *i* in reaction *j*. The linear equalities constrain the system to a steady state where fluxes into and out of every node are balanced. Nonequilibrium steady-states are enabled by including metabolite sources and sinks, collectively known as exchange reactions, at the boundary of the system with the environment. The inequalities arise from physicochemical constraints such as thermodynamics, as well as environmental constraints such as nutrient availability. Fluxes can be further constrained to the optimal value α∈ℝ of a biologically inspired linear objective$c\in {\mathbb{R}}^{n}$(Orth *et al.*, 2010).

Uniform sampling of constraint-based models (Thiele *et al.*, 2013) is a powerful tool for unbiased evaluation of the metabolic capabilities of biochemical networks (Lewis *et al.*, 2012). Most applications developed for this purpose (Megchelenbrink *et al.*, 2014; Saa and Nielsen, 2016; Thiele *et al.*, 2005) have been based on the artificial centering hit-and-run (ACHR) algorithm (Kaufman and Smith, 1998). ACHR is a non-Markovian process that is designed to ease exploration of a poorly structured set. However, it has some important drawbacks. Namely, it is not known whether it converges to the uniform distribution (Kaufman and Smith, 1998). Here, we present a Matlab implementation of coordinate hit-and-run with rounding (CHRR) that is compatible with the COnstraint-based Reconstruction and Analysis (COBRA) toolbox (Schellenberger *et al.*, 2011). A major difference with our approach is a preprocessing step which allows us to use a much simpler Markov chain to explore the set of metabolic flows. Rounding procedures have been used previously prior to sampling (De Martino *et al.*, 2015), but our approach achieves significant improvements for both the quality of the rounding produced and the efficiency of the sampling method (see Supplementary Methods Section S1). We gain inspiration and guidance from the current state-of-the-art theoretical results for high-dimensional sampling (Lovász and Vempala, 2006a,b), while making small modifications which drastically improve efficiency in practice. We compare the performance of CHRR with a comparable implementation of ACHR (Schellenberger *et al.*, 2011).

## 2 Implementation

CHRR consists of rounding followed by sampling (see Supplementary Methods Section S1 for details). To round an anisotropic polytope, we use a maximum volume ellipsoid algorithm (Zhang and Gao, 2001). The rounded polytyope is then sampled with a coordinate hit-and-run algorithm (Berbee *et al.*, 1987). Matlab (Mathworks, Natick, MA) implementations of these algorithms (Cousins and Vempala, 2016) were interfaced with the COBRA toolbox to permit sampling of any constraint-based metabolic model. The algorithmic inputs are a constraint-based metabolic model, that minimally includes *S*, *l*, *u* and *c* from Eq. 1, and parameters that control the length of the random walk and the sampling density (see Supplementary Tutorial).

## 3 Performance

When sampling the feasible set of a constraint-based model, it is important to run the sampling algorithm until the sampling distribution converges to a stationary distribution of fluxes over Ω. Otherwise, the sampling distribution is likely to be misrepresentative, leading to incorrect conclusions about the model (see Supplementary Figure). It is generally not empirically possible to verify convergence to the unknown distribution of fluxes over Ω. However, several measures exist that detect the absence of convergence to a stationary sampling distribution. Here, we used the potential scale reduction factor (Gelman *et al.*, 2013) as described in Supplementary Methods Section S2. For CHRR, it is known that the stationary distribution is the uniform distribution (Berbee *et al.*, 1987), but no such guarantees are known for ACHR.

**Fig. 1.**
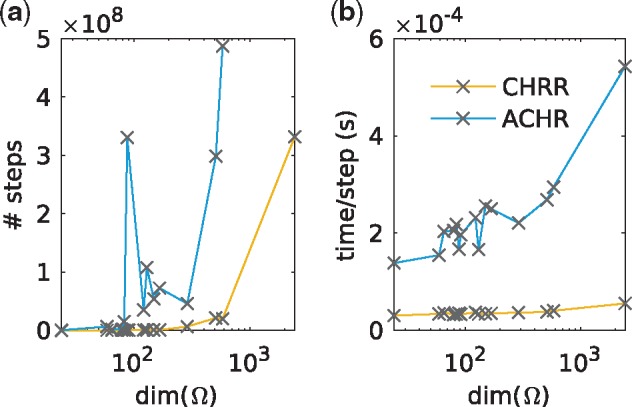
Convergence times. A comparison between the convergence times of CHRR and ACHR for 15 constraint-based models (see Supplementary Methods Section S3). (**a**) The number of steps of a random walk required for convergence to a stationary sampling distribution. ACHR did not converge in the maximum walk length of 10^9^ steps on two of the 15 models. These were the synechocystis model iJN678 (dim(Ω)=91) and the generic human model Recon 2 (dim(Ω)=2430). (**b**) Average time per step, computed out of 10^6^ steps

We compared the convergence time of CHRR to the COBRA toolbox implementation of ACHR (Fig. 1). We found that CHRR converged to a stationary sampling distribution in up to 730 times fewer steps than ACHR (Fig. 1a) on 15 models with dimensions ranging from 24 to 2430 (see Supplementary Methods Section S3). Moreover, each step of CHRR was up to 10 times faster than a step of ACHR (Fig. 1b). Each step of CHRR uses only a small number of arithmetic operations compared to ACHR, and this difference is only exaggerated as the dimension increases. Thus the improved scaling cannot be explained by programmatic differences between the two algorithms. These factors combined to give a 40–3500 fold speedup that tended to increase with model dimension.

## 4 Conclusions

Coordinate hit-and-run with rounding makes uniform sampling of genome-scale metabolic networks tractable and reliable. The compatibility of our implementation with the COBRA toolbox should facilitate widespread utilization by the constraint-based metabolic modelling community.

## Funding

HSH and IT were supported by the Luxembourg National Research Fund (FNR) through the National Centre of Excellence in Research (NCER) on Parkinson’s disease. BC and SV were supported in part by NSF awards CCF-1217793 and EAGER-1415498. RMTF was funded by the Interagency Modeling and Analysis Group, Multi-scale Modeling Consortium U01 awards from the National Institute of General Medical Sciences, award GM102098, and U.S. Department of Energy, Office of Science, Biological and Environmental Research Program, award DE-SC0010429.

*Conflict of Interest*: none declared.

## Supplementary Material

Supplementary Data
