# Supplementary material for: CHRR: coordinate hit-and-run with rounding for uniform sampling of constraint-based models
Source: Bioinformatics. 2017 Jan 31;33(11):1741–3. doi: 10.1093/bioinformatics/btx052 (PMC5447232; doi:10.1093/bioinformatics/btx052)
Supplement: Supplementary Data [file btx052_supp.zip › suppTutorial.pdf]

Uniform sampling

In this tutorial we will use Coordinate Hit-and-Run with Rounding (CHRR) [1] to uniformly sample a constraint-based model of the core metabolic network of *E. coli* [2].

A constraint-based metabolic model consists of a set of equalities and inequalities that define a convex polytope Ω of feasible flux vectors v,

$$\Omega = \{v | Sv = 0,\ l \leq v \leq u, c^T v = \alpha \},$$

where S is the $m \times n$ stoichiometric matrix, l and u are lower and upper bounds on fluxes, c is a linear objective and α is the solution to a flux balance analysis (FBA) problem [3].

CHRR consists of rounding followed by sampling. To round an anisotropic polytope, we use a maximum volume ellipsoid algorithm [4]. The rounded polytyope is then sampled with a coordinate hit-and-run algorithm [5].

Below is a high-level illustration of the process to uniformly sample a random metabolic flux vector v from the set Ω of all feasible metabolic fluxes (grey). **1)** Apply a rounding transformation T to Ω. The transformed set $\Omega' = T\Omega$ is such that its maximal inscribed ellipsoid (blue) approximates a unit ball. **2)** Take q steps of coordinate hit-and-run. At each step, i) pick a random coordinate direction e_i, and ii) move from current point $v'_k \in \Omega'$ to a random point $v'_{k+1} \in \Omega'$ along $v'_k + \alpha e_i \cap \Omega'$. **3)** Map samples back to the original space by applying the inverse transformation, e.g., $v_k = T^{-1} v'_k$.

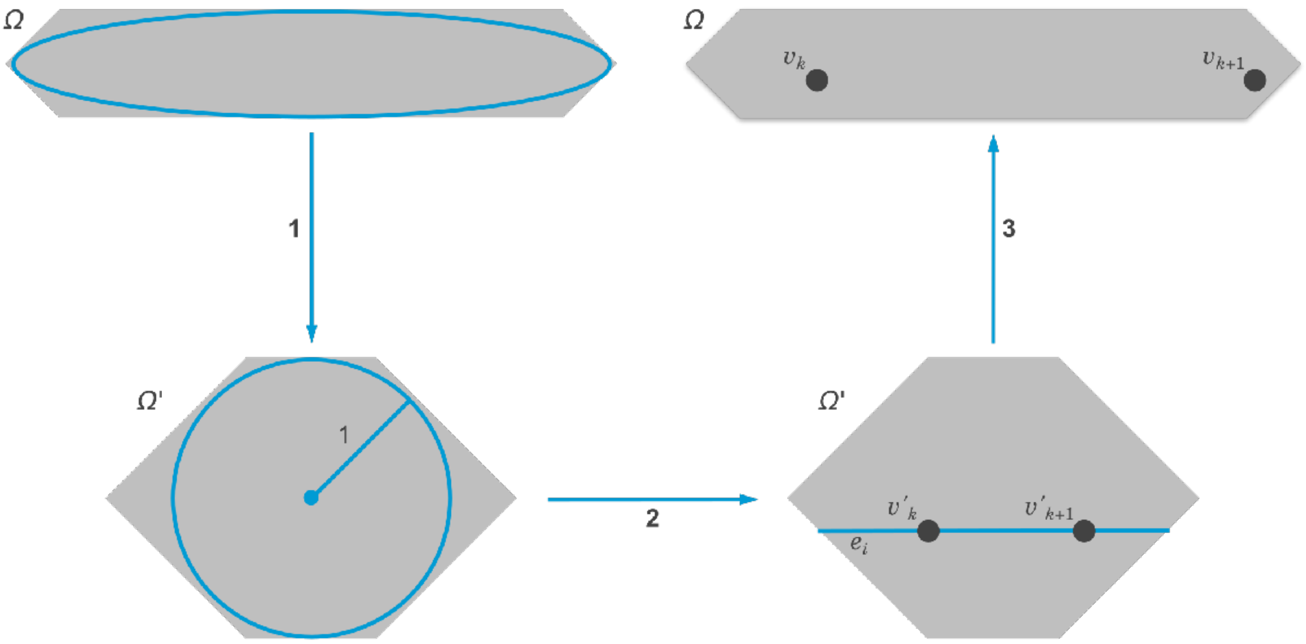

Modelling

We will model growth on glucose under aerobic and anaerobic conditions, following closely the flux balance analysis (FBA) tutorial published with [3].

We start by loading the model with published flux bounds and objective function (the biomass reaction). We set the maximum glucose uptake rate to 18.5 mmol/gDW/hr. To explore the entire space of feasible steady state fluxes we also remove the cellular objective.

```
load ../../testing/testSampling/Ecoli_core_model.mat
[m,n] = size(model.S);
model = changeRxnBounds(model,'EX_glc(e)',-18.5,'l');
model.c = 0*model.c; % linear objective
```

We allow unlimited oxygen uptake in the aerobic model and no oxygen uptake in the anaerobic model.

```
aerobic = changeRxnBounds(model,'EX_o2(e)',-1000,'l');
anaerobic = changeRxnBounds(model,'EX_o2(e)',0,'l');
```

Flux variability analysis

Flux variability analysis (FVA) returns the minimum and maximum possible flux through every reaction in a model.

```
startup % set user preferred LP solver etc.

[minAer,maxAer] = fluxVariability(aerobic);
```

```
Starting parallel pool (parpool) using the 'local' profile ... connected to 4 workers.
```

```
[minAna,maxAna] = fluxVariability(anaerobic);
```

FVA predicts faster maximal growth under aerobic than anaerobic conditions.

```
bm = 'Biomass_Ecoli_core_N(w/GAM)-Nmet2'; % biomass reaction identifier
ibm = find(ismember(model.rxns,bm)); % colunn index of biomass reaction
fprintf('Max. aerobic growth: %.4f/h.\n', maxAer(ibm));
```

```
Max. aerobic growth: 1.6531/h.
```

```
fprintf('Max. anaerobic growth: %.4f/h.\n\n', maxAna(ibm));
```

```
Max. anaerobic growth: 0.4706/h.
```

An overall comparison of the FVA results can be obtained by computing the Jaccard index for each reaction. The Jaccard index is here defined as the ratio between the intersection and union of the flux ranges in the aerobic and anaerobic models. A Jaccard index of 0 indicates completely disjoint flux ranges and a Jaccard index of 1 indicates completely overlapping flux ranges. The mean Jaccard index gives an indication of the overall similarity between the models.

```
J = fvaJaccardIndex([minAer minAna],[maxAer,maxAna]);
fprintf('Mean Jaccard index = %.4f.\n',mean(J));
```

```
Mean Jaccard index = 0.4654.
```

To visualise the FVA results, we plot the flux ranges as errorbars, with reactions sorted by the Jaccard index.

```
E = [(maxAer - minAer)/2 (maxAna - minAna)/2];
Y = [minAer minAna] + E;
X = [(1:length(Y)) - 0.1; (1:length(Y)) + 0.1]';

[~,xj] = sort(J);

f1 = figure;
errorbar(X,Y(xj,:),E(xj,:),'linestyle','none','linewidth',2,'capsize',0);
set(gca,'xlim',[0 length(Y)+1])
legend('Aeorobic','Anaerobic','location','northoutside','orientation','horizontal')
xlabel('Reaction')
ylabel('Flux range (mmol/gDW/h)')

yyaxis right
plot(J(xj))
ylabel('Jaccard index')
```

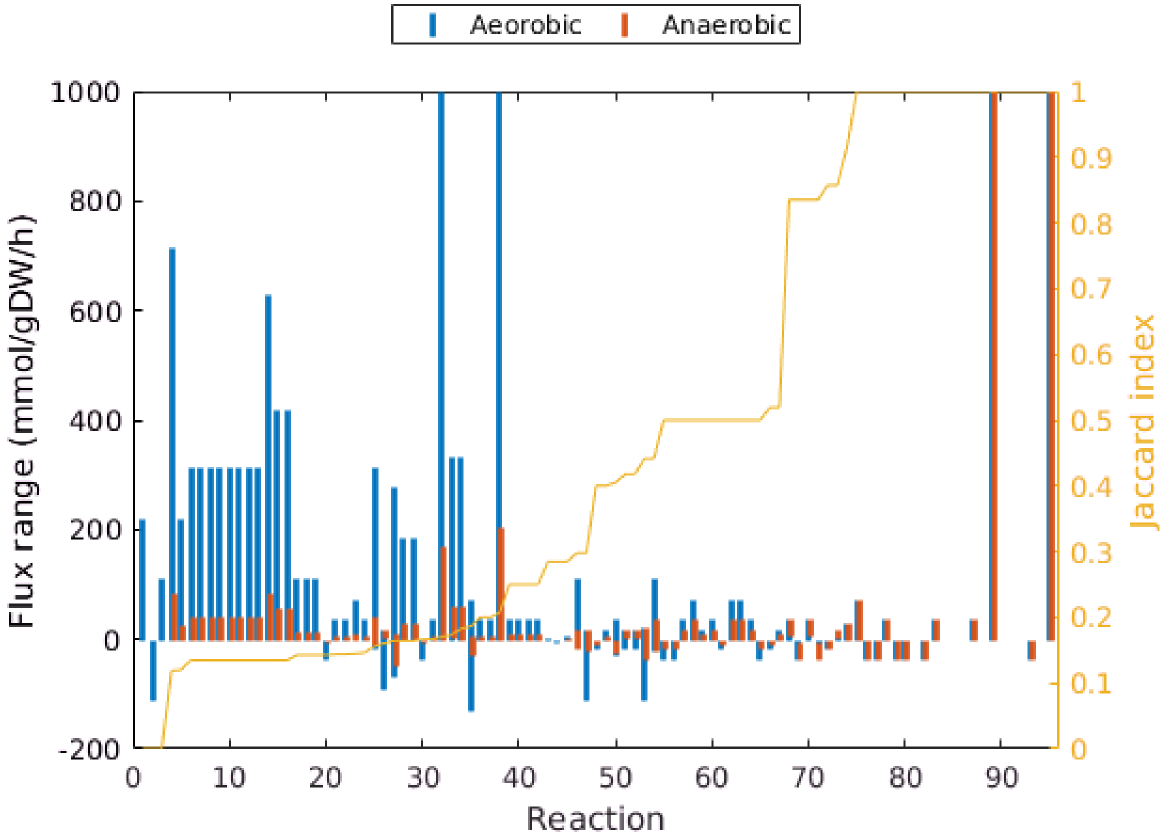

Sampling

CHRR can be called via either the function chrrSampler, or sampleCbModel. We will use the former route here. Type "`help sampleCbModel`" to learn about the second route.

The main inputs to chrrSampler are a COBRA model structure and parameters that control the sampling density (nSkip) and the number of samples (nSamples). The total length of the random walk is nSkip*nSamples. The time it takes to run the sampler depends on the total length of the random walk and the size of the model [1]. However, using sampling parameters that are too small will lead to invalid sampling distributions, e.g.,

```
nSkip = 1;
nSamples = 100;
```

With these parameter settings, it should only take a few seconds to sample the two E. coli core models.

An additional on/off parameter (toRound) controls whether or not the polytope is rounded. Rounding large models can be slow but is strongly recommended for the first round of sampling. Below we show how to get around this step in subsequent rounds.

```
toRound = 1;
```

To sample the aerobic and anaerobic E. coli core models, run,

```
[X1_aer,P_aer] = chrrSampler(aerobic,nSkip,nSamples,toRound);
```

```
Checking for width 0 facets...
Starting parallel pool (parpool) using the 'local' profile ... connected to 4 workers.
Currently (P.A, P.b) are in 95 dimensions
Warning: Rank deficient, rank = 72, tol =  1.058337e-10.

Now in 23 dimensions after restricting
Removed 18 zero rows
Rounding...

  Residuals:   Primal     Dual    Duality  logdet(E)
  --------------------------------------------------
  iter  10    4.5e+00   4.7e-01   2.1e-01  2.709e+01
  iter  20    2.2e+02   1.7e-02   1.7e-02  5.758e+01
  iter  30    6.3e+02   2.4e-03   2.4e-03  6.189e+01
  iter  40    1.4e+00   4.5e-06   4.7e-06  6.261e+01
  Converged!
  [m, n] = [172, 23]
  CPU time: 0.29 seconds
ans = 1.5610e+27
  Residuals:   Primal     Dual    Duality  logdet(E)
  --------------------------------------------------
  Converged!
  [m, n] = [172, 23]
  CPU time: 0.07 seconds
Trying to find a point inside the convex body...
Final polytope is in 23 dimensions.
```

```
[X1_ana,P_ana] = chrrSampler(anaerobic,nSkip,nSamples,toRound);
```

```
Checking for width 0 facets...
Currently (P.A, P.b) are in 95 dimensions
Warning: Rank deficient, rank = 73, tol =  1.076377e-10.

Now in 22 dimensions after restricting
Removed 24 zero rows
Rounding...

  Residuals:   Primal     Dual    Duality  logdet(E)
  --------------------------------------------------
  iter  10    6.5e+00   7.7e-01   2.4e-01  6.856e+00
  iter  20    1.0e+01   1.6e-01   9.7e-02  2.523e+01
  iter  30    1.1e+03   5.9e-03   5.2e-03  3.561e+01
  iter  40    4.4e+00   2.0e-05   2.0e-05  3.622e+01
  Converged!
  [m, n] = [166, 22]
  CPU time: 0.3 seconds
ans = 5.3847e+15
  Residuals:   Primal     Dual    Duality  logdet(E)
```

```
  --------------------------------------------------
  Converged!
  [m, n] = [166, 22]
  CPU time: 0.03 seconds
Trying to find a point inside the convex body...
Final polytope is in 22 dimensions.
```

The sampler outputs the sampled flux distributions (X_aer and X_ana) and the rounded polytope (P_aer and P_ana). Histograms of sampled biomass reaction flux show that the models are severly undersampled, as evidenced by the presence of multiple sharp peaks.

```
nbins = 15;
[yAer,xAer] = hist(X1_aer(ibm,:),nbins);
[yAna,xAna] = hist(X1_ana(ibm,:),nbins);

f2 = figure;
plot(xAer,yAer,xAna,yAna);
legend('Aeorobic','Anaerobic')
xlabel('Flux (mmol/gDW/h)')
ylabel('# samples')
```

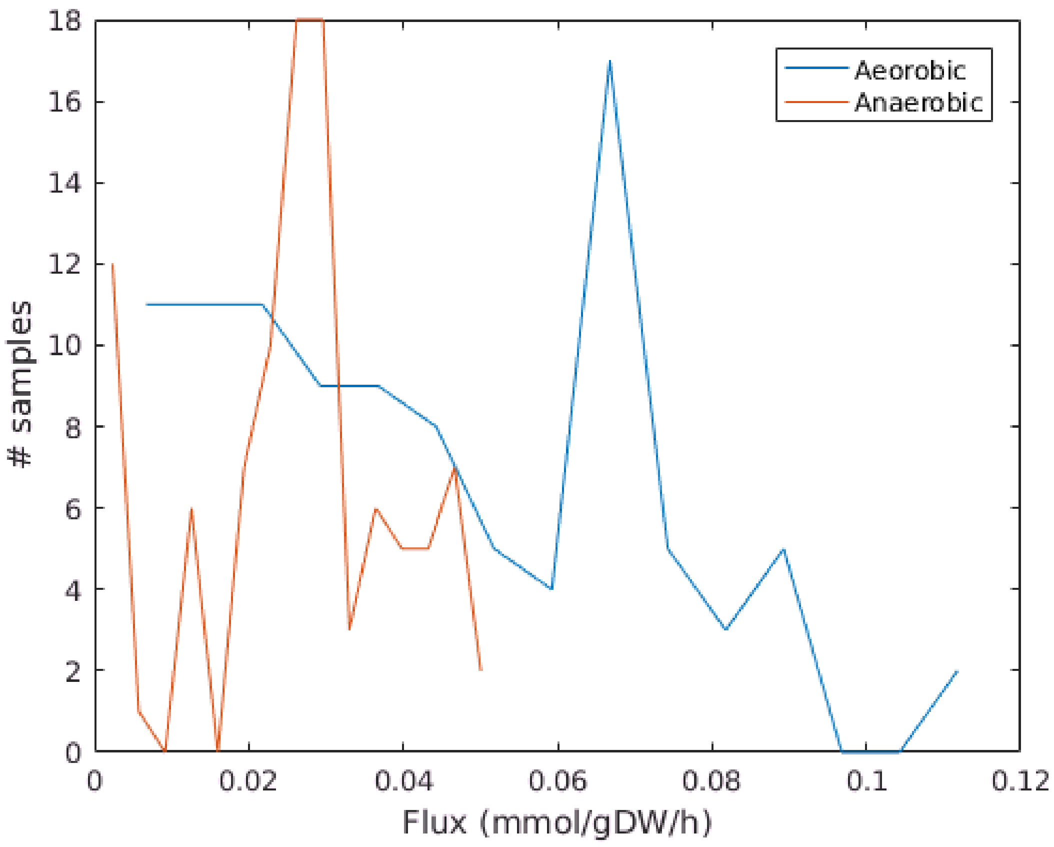

Undersampling results from selecting too small sampling parameters. The appropriate parameter values depend on the dimension of the polytope Ω defined by the model constraints (see intro). One rule of thumb says to set $\mathrm{nSkip} = 8 * \dim(\Omega)^2$ to ensure statistical independence of samples. The random walk should be long enough to ensure convergence to a stationary sampling distribution [1].

The dimension of the polytope for E. coli core is $\dim(\Omega) = 22$ for the aerobic model and $\dim(\Omega) = 21$ for the anaerobic model. A good choice of sampling parameters is,

```
nSkip = 5e3;
nSamples = 1e3;
```

With these parameter settings, it should take around 2.5 minutes to sample each E. coli core model. This time, we can avoid the rounding step by inputting the rounded polytope from the previous round of sampling.

```
toRound = 0;
X2_aer = chrrSampler(aerobic,nSkip,nSamples,toRound,P_aer);
X2_ana = chrrSampler(anaerobic,nSkip,nSamples,toRound,P_ana);
```

The converged sampling distributions for the biomass reaction are much smoother, with a single peak at zero flux.

```
nbins = 15;
[yAer,xAer] = hist(X2_aer(ibm,:),nbins);
[yAna,xAna] = hist(X2_ana(ibm,:),nbins);

f3 = figure;
p1 = plot(xAer,yAer,xAna,yAna);
legend('Aeorobic','Anaerobic')
xlabel('Flux (mmol/gDW/h)')
ylabel('# samples')
```

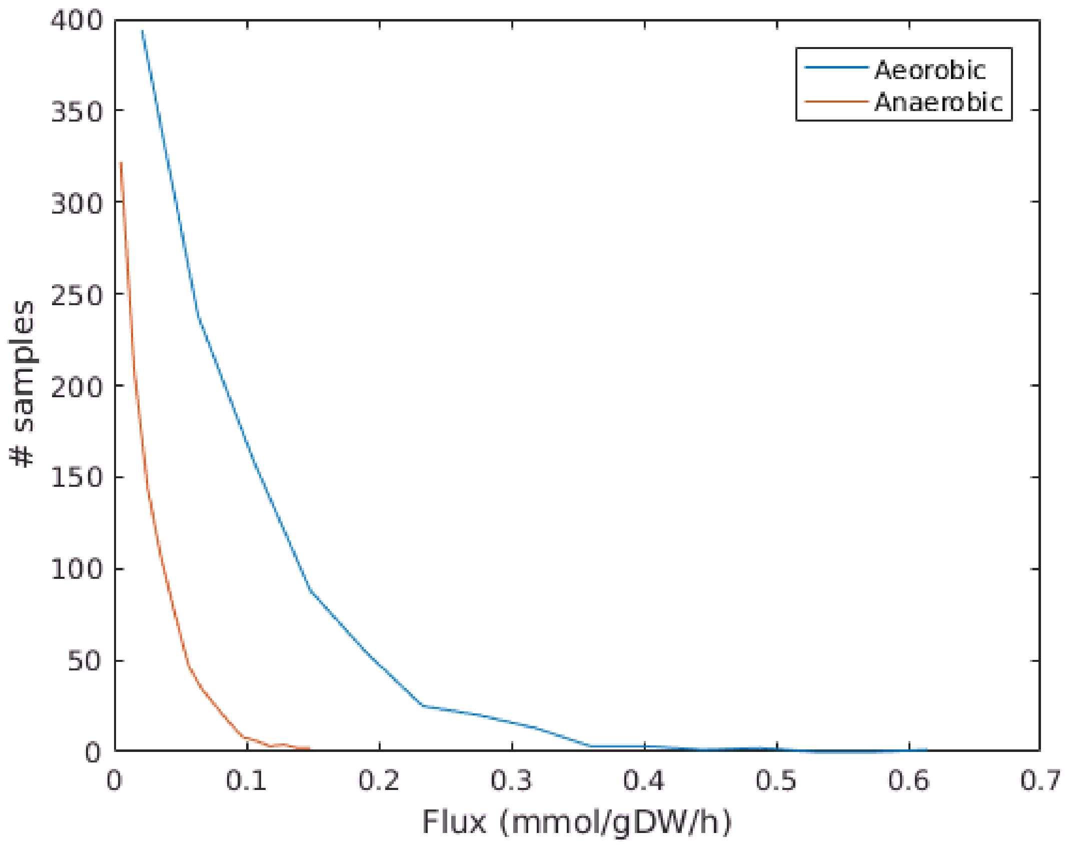

Adding the FVA results to the plot shows that the sampling distributions give more detailed information about the differences between the two models. In particular we see that the flux minima and maxima are not equally probable. The number of samples from both the aerobic and anaerobic models peaks at the minum flux of zero, and decreases monotonically towards the maximum. It decreases more slowly in the aerobic model, indicating that higher biomass flux is more probable under aerobic conditions. It is interesting to see that maximum growth is highly improbable in both models.

```
ylim = get(gca,'ylim');
cAer = get(p1(1),'color');
cAna = get(p1(2),'color');

hold on
p2 = plot([minAer(ibm) minAer(ibm)],ylim,'--',[maxAer(ibm) maxAer(ibm)],ylim,'--');
set(p2,'color',cAer)
p3 = plot([minAna(ibm) minAna(ibm)],ylim,'--',[maxAna(ibm) maxAna(ibm)],ylim,'--');
set(p3,'color',cAna)
hold off
```

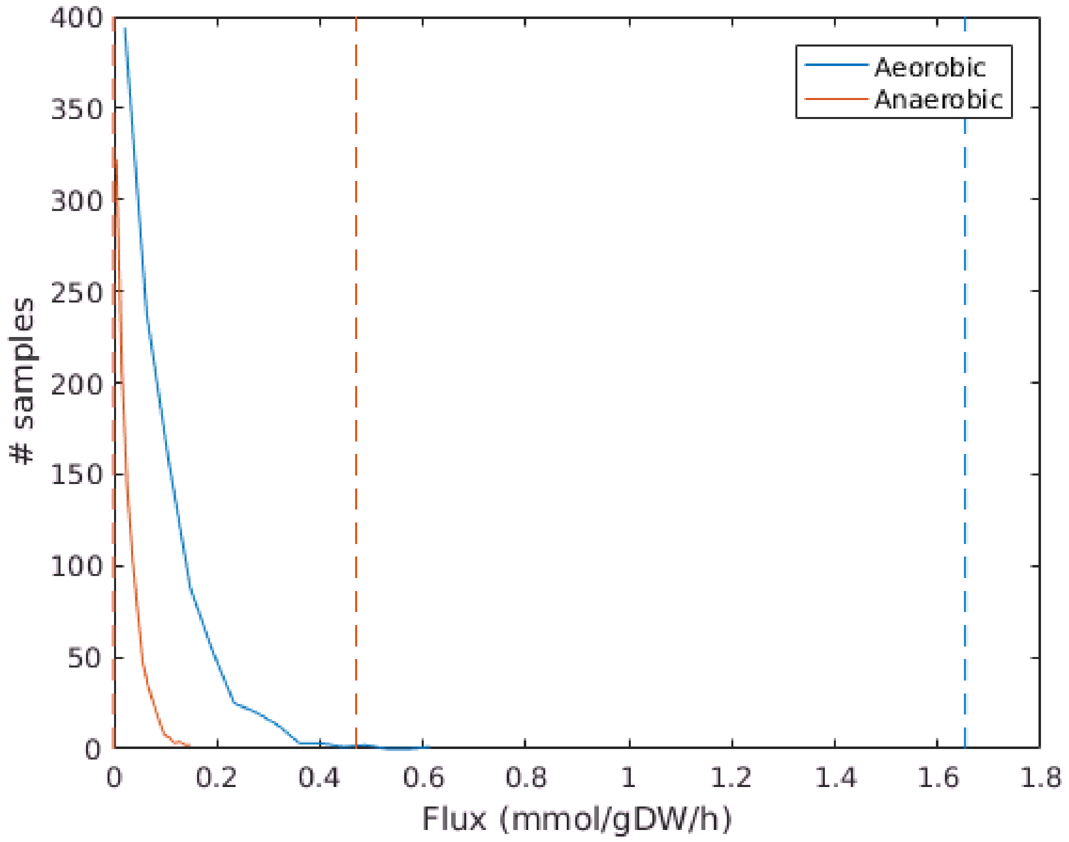

Finally, plotting sampling distributions for six randomly selected E. coli core reactions shows how oxygen availability affects a variety of metabolic pathways.

```
f4 = figure;
position = get(f4,'position');
set(f4,'units','centimeters','position',[position(1) position(2) 18 27])

ridx = randi(n,1,6);
```

```
for i = ridx
    nbins = 15;
    [yAer,xAer] = hist(X2_aer(i,:),nbins);
    [yAna,xAna] = hist(X2_ana(i,:),nbins);

    subplot(3,2,find(ridx==i))
    h1 = plot(xAer,yAer,xAna,yAna);
    xlabel('Flux (mmol/gDW/h)')
    ylabel('# samples')
    title(sprintf('%s (%s)',model.subSystems{i},model.rxns{i}),'FontWeight','normal')

    if find(ridx==i)==1
        legend('Aeorobic','Anaerobic')
    end

    ylim = get(gca,'ylim');

    hold on
    h2 = plot([minAer(i) minAer(i)],ylim,'--',[maxAer(i) maxAer(i)],ylim,'--');
    set(h2,'color',cAer)
    h3 = plot([minAna(i) minAna(i)],ylim,'--',[maxAna(i) maxAna(i)],ylim,'--');
    set(h3,'color',cAna)
    hold off
end
```

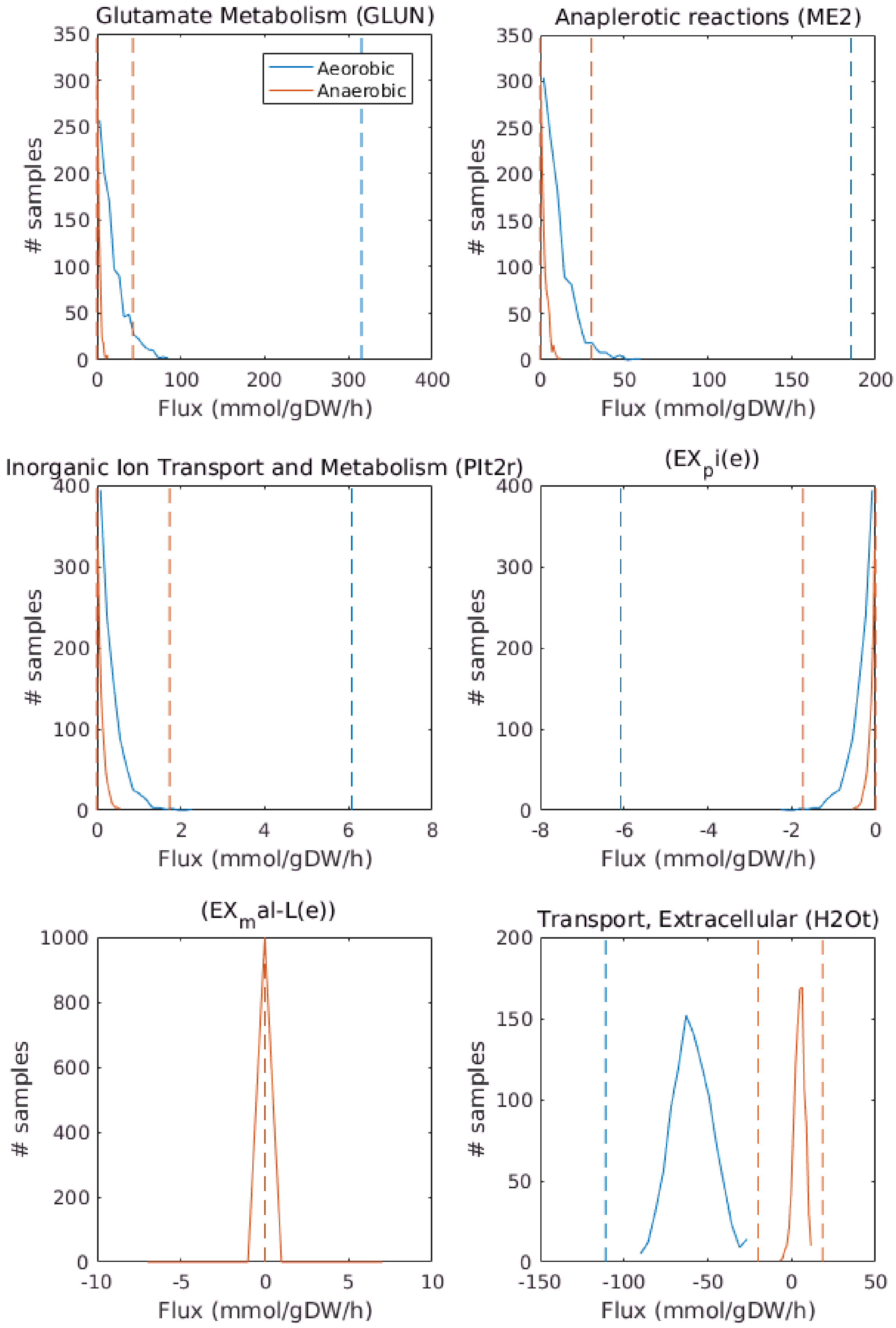
Glutamate Metabolism (GLUN)
Aeorobic
Anaerobic
samples
Flux (mmol/gDW/h)
Anaplerotic reactions (ME2)
samples
Flux (mmol/gDW/h)
Inorganic Ion Transport and Metabolism (PIt2r)
samples
Flux (mmol/gDW/h)
(EX_pi(e))
samples
Flux (mmol/gDW/h)
(EX_mal-L(e))
samples
Flux (mmol/gDW/h)
Transport, Extracellular (H2Ot)
samples
Flux (mmol/gDW/h)

References

[1] Haraldsdóttir, H. S., Cousins, B., Thiele, I., Fleming, R.M.T., and Vempala, S. (2016). CHRR: coordinate hit-and-run with rounding for uniform sampling of constraint-based metabolic models. Submitted.

[2] Orth, J. D., Palsson, B. Ø., and Fleming, R. M. T. (2010). Reconstruction and use of microbial metabolic networks: the core Escherichia coli metabolic model as an educational guide. EcoSal Plus, 1(10).

[3] Orth, J. D., Thiele I., and Palsson, B. Ø. (2010). What is flux balance analysis? Nat. Biotechnol., 28(3), 245–248.

[4] Zhang, Y. and Gao, L. (2001). On Numerical Solution of the Maximum Volume Ellipsoid Problem. SIAM J. Optimiz., 14(1), 53–76.

[5] Berbee, H. C. P., Boender, C. G. E., Rinnooy Ran, A. H. G., Scheffer, C. L., Smith, R. L., Telgen, J. (1987). Hit-and-run algorithms for the identification of nonredundant linear inequalities. Math. Programming, 37(2), 184–207.
