# Supplementary material for: CHRR: coordinate hit-and-run with rounding for uniform sampling of constraint-based models
Source: Bioinformatics. 2017 Jan 31;33(11):1741–3. doi: 10.1093/bioinformatics/btx052 (PMC5447232; doi:10.1093/bioinformatics/btx052)
Supplement: Supplementary Data [file btx052_supp.zip › suppFigure.pdf]

Supplementary Figure

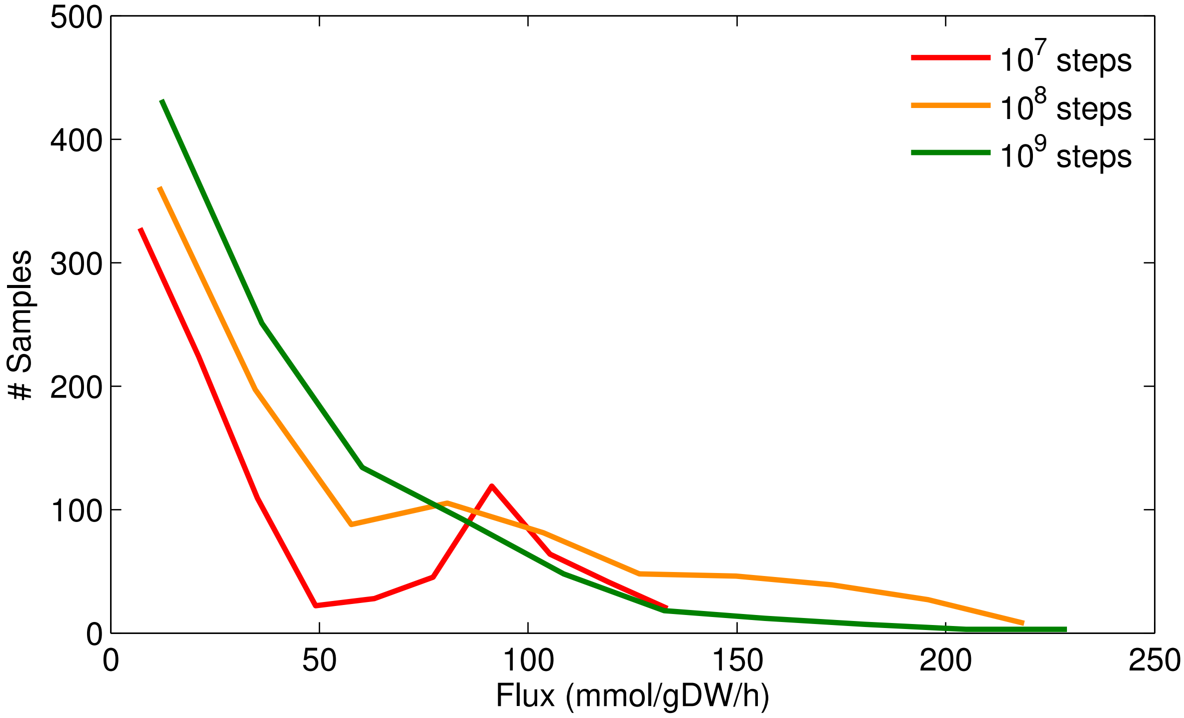

Fig. 1: **Convergence to a stationary sampling distribution.** The marginal distribution of thioredoxin reductace flux samples in a constraint-based model of human metabolism (Recon 2 [1]). CHRR converged to a stationary sampling distribution in 1.5724×10^8 steps. The post-convergence sampling distribution (green line) shows that flux probability decreases monotonically. The pre-convergence sampling distributions (red and orange lines) would lead to the incorrect conclusion that probability peaks at more than one flux value. Information about the thioredoxin reductase reaction is available in the Virtual Metabolic Human (VMH) database at https://vmh.uni.lu/#reaction/TRDR2.

References

[1] Thiele, I., Swainston, N., Fleming, R. M. T., Hoppe, A., Sahoo, S., Aurich, M. K., Haraldsdóttir, H., Mo, M. L., Rolfsson, O., Stobbe, M. D., Thorleifsson, S. G., Agren, R., Bölling, C., Bordel, S., Chavali, A. K., Dobson, P., Dunn, W. B., Endler, L., Hala, D., Hucka, M., Hull, D., Jameson, D., Jamshidi, N., Jonsson, J. J., Juty, N., Keating, S., Nookaew, I., Le Novère, N., Malys, N., Mazein, A., Papin, J. A., Price, N. D., Selkov Sr, E., Sigurdsson, M. I., Simeonidis, E., Sonnenschein, N., Smallbone, K., Sorokin, A., van Beek, J. H. G. M., Weichart, D., Goryanin, I., Nielsen, J., Westerhoff, H. V., Kell, D. B., Mendes, P., and Palsson, B. Ø. (2013). A community-driven global reconstruction of human metabolism. *Nat. Biotechnol.*, **31**(5), 419–425.
