# Supplementary material for: CHRR: coordinate hit-and-run with rounding for uniform sampling of constraint-based models
Source: Bioinformatics. 2017 Jan 31;33(11):1741–3. doi: 10.1093/bioinformatics/btx052 (PMC5447232; doi:10.1093/bioinformatics/btx052)
Supplement: Supplementary Data [file btx052_supp.zip › suppMethods.pdf]

Supplementary Methods

1 Implementation

Here we outline the two main components of our sampling algorithm. The algorithm consists of a rounding preprocessing step via computation of the maximum volume ellipsoid, followed by generating samples in the rounded space using coordinate hit-and-run (CHR). In the descriptions below, when we refer to a polytope $P = \{x \in \mathbb{R}^n | Ax \leq b\}$, this can be thought of as the set of feasible flux vectors. Also, the O^*notation suppresses logarithmic factors and dependence on error parameters to emphasize the dependence on the dimension.

1.1 Rounding

The efficiency of the random walks have a strong connection to the roundedness of the polytope. For the random walks we consider here, if the walk is run on an unrounded polytope, then the number of steps until convergence can be arbitrarily high. However, there are rigorous guarantees of convergence once the polytope is rounded.

There are two different notions of roundedness we can consider: well-roundedness and isotropy. For a polytope $P = \{x \in \mathbb{R}^n | Ax \leq b\}$, we say that P is R-rounded if $B_n \subseteq P \subseteq R \cdot B_n$, i.e. the body P is sandwiched between a ball of radius 1 and a ball of radius R. Further we say that P is well-rounded if $R = O^*(\sqrt{n})$. If P is well-rounded, then there are random walks which are known to converge after $O^*(n^3)$ steps. Two such random walks are the ball walk [11, 4] and hit-and-run [13, 14]. Each of these walks has mixing time $O^*(R^2n^2)$. Therefore, it is crucial for efficiency that the rounding procedure achieves R close to $\sqrt{n}$.

One approach to achieve well-roundedness is by putting P into isotropic position. We say that a polytope P is in isotropic position if its center of mass is the origin and its covariance matrix is the identity, i.e., $E_{X\sim P}(X) = 0$ and $E_{X\sim P}(X^TX) = I$. It is well known that for every polytope P, there exists an affine transformation T such that $T \cdot P$ is in isotropic position. If P is isotropic, then it is essentially well-rounded. More precisely, if P is isotropic, then it contains the unit ball [27, 10], and we can restrict P to a ball of radius $O^*(\sqrt{n})$ to only ignore a negligible fraction of its mass [16].

Algorithmically, however, putting P into isotropic position can be quite expensive. A natural approach is to generate random samples from P, then apply an affine transformation so that the samples have zero mean and identity covariance matrix. This approach can be iterated until the body is in approximate isotropic position [15, 5]. While this approach guarantees the body will be well-rounded, it is computationally expensive and is impractical to scale to genome scale biological networks.

We instead find that an alternative method of finding the maximum volume ellipsoid in the polytope P is orders of magnitude faster. For our implementation, we place the polytope in John's position via computing the maximum volume inscribed ellipsoid and transforming it to the unit ball (Figure 1). A body is said to be in John's position if the maximal inscribed ellipsoid is the unit ball. A classic result shows that a body in John's position is n-rounded [9]. In the worst case, this is worse by a factor of $O^*(\sqrt{n})$ from isotropy or well-roundedness. Nevertheless, in practice we observe that rounding via the maximum volume ellipsoid has comparable efficiency to rounding via isotropic position.

1.2 Coordinate hit-and-run

To generate uniform random points from a polytope $P = \{x \in \mathbb{R}^n | Ax \leq b\}$, we use the coordinate hit-and-run (CHR) Markov chain. The walk is guaranteed to converge to the uniform distribution [2].

A close variant of CHR is known as hit-and-run. The walk is equivalent to CHR, except instead of picking a random coordinate direction, it selects a uniformly random direction from the current point. For a well-rounded polytope $P \subseteq \mathbb{R}^n$, hit-and-run is guaranteed to converge to the uniform distribution after $O^*(n^3)$ steps [14]. The mixing time can be expressed more generally as $O^*(\frac{R}{r^2}^2 \cdot n^2)$ for a polytope P such that $r \cdot B_n \subseteq P \subseteq R \cdot B_n$. Hit-and-run was used previously in the work of [17] for sampling metabolic networks.

While no such convergence guarantees are known for CHR, it experimentally appears to converge in the same number of steps as hit-and-run for the polytopes we consider. Importantly, each step of CHR can be implemented a factor of n faster than a step of hit-and-run. So while there are no rigorous guarantees, we use CHR because experimentally it substantially outperforms hit-and-run.

We run CHR in the rounded polytope; to recover the samples in the original space, we simply apply the inverse transformation (Figure 1).

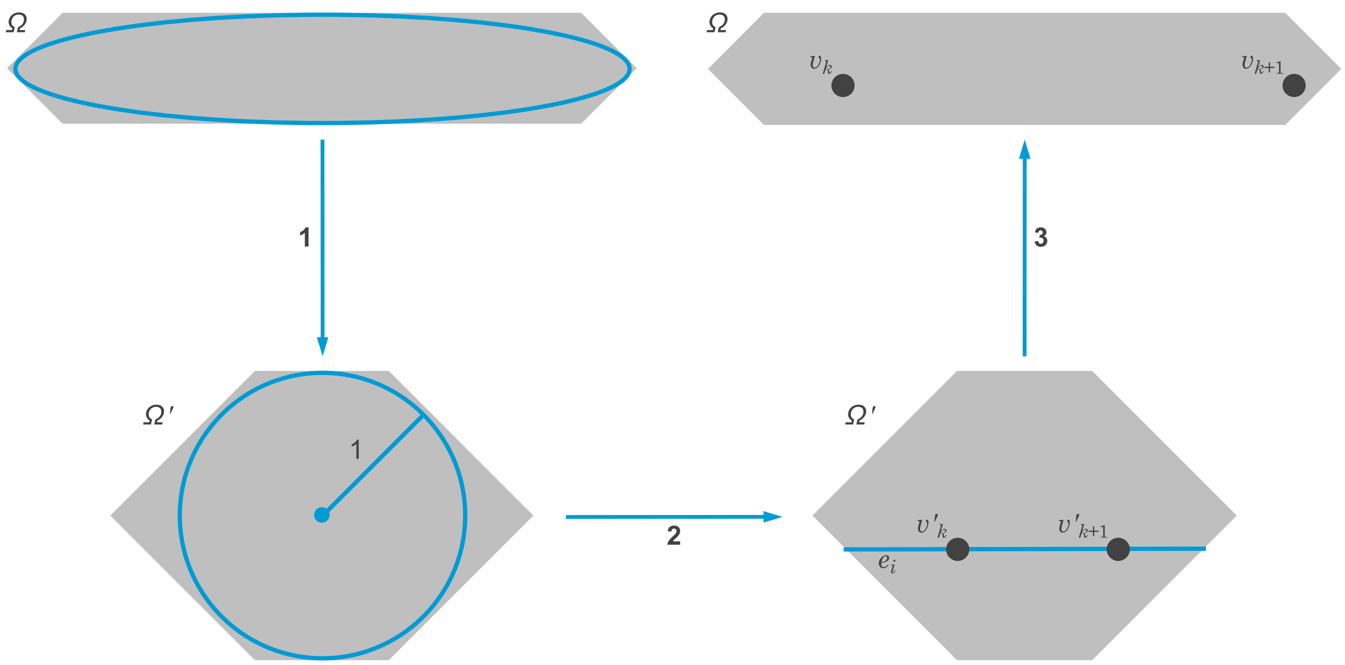

Fig. 1: **Coordinate hit-and-run with rounding.** A high-level illustration of the process to uniformly sample a random metabolic flux vector v from the set Ω of all feasible metabolic fluxes (grey). 1) Apply a rounding transformation T to Ω. The transformed set $\Omega' = T\Omega$ is such that its maximal inscribed ellipsoid (blue) approximates a unit ball. 2) Take q steps of coordinate hit-and-run. At each step, i) pick a random coordinate direction e_i, and ii) move from current point $v'_k \in \Omega'$ to a random point $v'_{k+1} \in \Omega'$ along $v'_k + \alpha e_i \cap \Omega'$. 3) Map samples back to the original space by applying the inverse transformation, e.g., $v_k = T^{-1} v'_k$.

2 Convergence test

Suppose we have a sequence of points $X_1, X_2, \ldots, X_k$ from a convex body $P = \{x \in \mathbb{R}^n | Ax \leq b\}$, where each X_i is generated from running CHR for t steps in K. If CHR has converged, then each X_i should be approximately uniform from K. While exactly testing uniformity of the set of X_i's is computationally intractable, there are a variety of statistical tests which can detect the absence of convergence.

In our experimental tests, we declare convergence once the stream of points passes a chosen set of statistical tests. It is possible that convergence is declared prematurely, and the points X_i are very far from uniform. This fact serves as motivation to theoretically ground the high-dimensional sampling algorithm as much as possible. For CHR, we are at least guaranteed that the process will converge to uniform given enough steps, and for hit-and-run, we have precise bounds on the number of steps until convergence.

Convergence to a stationary sampling distribution was determined empirically by computing the potential scale reduction factor (PSRF) [8] of the marginal sampling distributions for all reactions. To compute the PSRF, the set of samples is divided in half and each half is considered a separate chain. The variance of the samples within each chain is computed as well as the variance of the samples between the chains. A low PSRF indicates that these variances are close in magnitude. The PSRF was computed with the MCMC Diagnostics Toolbox for Matlab [25]. An algorithm was said to have converged when the PSRF of all marginals was stable below 1.1.

3 Models

Computational experiments were run on the 15 constraint-based models listed in Table 1. All models were downloaded as a Matlab structure in the standard COBRA toolbox format [26]. Recon 2 was downloaded from the Virtual Metabolic Human (VMH) database at https://vmh.uni.lu/. All other models were downloaded from the BiGG Models database [12] at http://bigg.ucsd.edu/. Computational experiments were run with published

flux bounds. The objective function was set to zero to sample the largest possible space. We note that the algorithm can also uniformly sample solutions that achieve a specified objective function value.

Tab. 1: **Constraint-based metabolic models.**

Name	Metabolites (m)	Reactions (n)	Dimension ($d = dim(\Omega)$)	Reference
e_coli_core	72	95	24	[23]
iLJ478	570	652	59	[31]
iIT341	485	554	65	[28]
iAF692	628	690	79	[6]
iSB619	655	743	83	[1]
iHN637	698	785	88	[19]
iJN678	795	863	91	[21]
iJN746	909	1056	122	[20]
iAB_RBC_283	342	469	130	[3]
iAF987	1109	1285	149	[7]
iYO844	991	1250	167	[22]
iAT_PLT_636	738	1008	289	[30]
iSDY_1059	1890	2540	509	[18]
iJO1366	1805	2583	582	[24]
Recon2_v04	5063	7440	2430	[29]

References

[1] Becker, S. A. and Palsson, B. Ø. (2005). Genome-scale reconstruction of the metabolic network in Staphylococcus aureus N315: an initial draft to the two-dimensional annotation. *BMC microbiol.*, **5**, 8.

[2] Berbee, H. C. P., Boender, C. G. E., Rinnooy Kan, A. H. G., Scheffer, C. L., Smith, R. L., and Telgen, J. (1987). Hit-and-run algorithms for the identification of nonredundant linear inequalities. *Math. Programming*, **37**(2), 184–207.

[3] Bordbar, A., Jamshidi, N., and Palsson, B. Ø. (2011). iAB-RBC-283: A proteomically derived knowledge-base of erythrocyte metabolism that can be used to simulate its physiological and patho-physiological states. *BMC Syst. Biol.*, **5**(1), 110.

[4] Cousins, B. and Vempala, S. (2015). Bypassing KLS: Gaussian cooling and an $O^*(n^3)$ volume algorithm. In *STOC*, pages 539–548.

[5] Cousins, B. and Vempala, S. (2016). A practical volume algorithm. *Math. Prog. Computation*, **8**(2), 133–160.

[6] Feist, A. M., Scholten, J. C. M., Palsson, B. Ø., Brockman, F. J., and Ideker, T. (2006). Modeling methanogenesis with a genome-scale metabolic reconstruction of Methanosarcina barkeri. *Mol. Syst. Biol.*, **2**, 2006.0004.

[7] Feist, A. M., Nagarajan, H., Rotaru, A.-E., Tremblay, P.-L., Zhang, T., Nevin, K. P., Lovley, D. R., and Zengler, K. (2014). Constraint-based modeling of carbon fixation and the energetics of electron transfer in Geobacter metallireducens. *PLoS comput. biol.*, **10**(4), e1003575.

[8] Gelman, A., Carlin, J. B., Stern, H. S., Dunson, D. B., Vehtari, A., and Rubin, D. B. (2013). *Bayesian Data Analysis*. Chapman and Hall/CRC, London, UK, 3rd edition.

[9] John, F. (1948). Extremum problems with inequalities as subsidiary conditions. In *Studies and Essays Presented to R. Courant on his 60th Birthday, January 8, 1948*, pages 187–204. Interscience Publishers, Inc., New York, N. Y.

[10] Kannan, R., Lovász, L., and Simonovits, M. (1995). Isoperimetric problems for convex bodies and a localization lemama. *Discrete Comput. Geom.*, **13**(3), 541–559.

[11] Kannan, R., Lovász, L., and Simonovits, M. (1997). Random walks and an $O^*(n^5)$ volume algorithm for convex bodies. *Random Struct. and Algor.*, **11**(1), 1–50.

[12] King, Z. A., Lu, J., Dräger, A., Miller, P., Federowicz, S., Lerman, J. A., Ebrahim, A., Palsson, B. O., and Lewis, N. E. (2016). BiGG Models: A platform for integrating, standardizing and sharing genome-scale models. *Nucleic Acids Res.*, **44**(Database issue), D515–D522.

[13] Lovász, L. (1998). Hit-and-run mixes fast. *Math. Prog*, **86**(3), 443–461.

[14] Lovász, L. and Vempala, S. (2006a). Hit-and-run from a corner. *SIAM J. Computing*, **35**(4), 985–1005.

[15] Lovász, L. and Vempala, S. (2006b). Simulated annealing in convex bodies and an $O^*(n^4)$ volume algorithm. *J. Comput. Syst. Sci.*, **72**(2), 392–417.

[16] Lovász, L. and Vempala, S. (2007). The geometry of logconcave functions and sampling algorithms. *Random Struct. Algorithms*, **30**(3), 307–358.

[17] Martino, D. D., Mori, M., and Parisi, V. (2015). Uniform Sampling of Steady States in Metabolic Networks: Heterogeneous Scales and Rounding. *PLOS ONE*, **10**(4), e0122670.

[18] Monk, J. M., Charusanti, P., Aziz, R. K., Lerman, J. A., Premyodhin, N., Orth, J. D., Feist, A. M., and Palsson, B. Ø. (2013). Genome-scale metabolic reconstructions of multiple Escherichia coli strains highlight strain-specific adaptations to nutritional environments. *PNAS*, **110**(50), 20338–20343.

[19] Nagarajan, H., Sahin, M., Nogales, J., Latif, H., Lovley, D. R., Ebrahim, A., and Zengler, K. (2013). Characterizing acetogenic metabolism using a genome-scale metabolic reconstruction of Clostridium ljungdahlii. *Microb. Cell. Fact.*, **12**, 118.

[20] Nogales, J., Palsson, B. Ø., and Thiele, I. (2008). A genome-scale metabolic reconstruction of Pseudomonas putida KT2440: iJN746 as a cell factory. *BMC Syst. Biol.*, **2**, 79.

[21] Nogales, J., Gudmundsson, S., Knight, E. M., Palsson, B. O., and Thiele, I. (2012). Detailing the optimality of photosynthesis in cyanobacteria through systems biology analysis. *PNAS*, **109**(7), 2678–2683.

[22] Oh, Y.-K., Palsson, B. O., Park, S. M., Schilling, C. H., and Mahadevan, R. (2007). Genome-scale reconstruction of metabolic network in Bacillus subtilis based on high-throughput phenotyping and gene essentiality data. *J. Biol. Chem.*, **282**(39), 28791–28799.

[23] Orth, J. D., Palsson, B. Ø., and Fleming, R. M. T. (2010). Reconstruction and Use of Microbial Metabolic Networks: the Core Escherichia coli Metabolic Model as an Educational Guide. *EcoSal Plus*, **1**(10).

[24] Orth, J. D., Conrad, T. M., Na, J., Lerman, J. A., Nam, H., Feist, A. M., and Palsson, B. Ø. (2011). A comprehensive genome-scale reconstruction of Escherichia coli metabolism–2011. *Mol. Syst. Biol.*, **7**, 535.

[25] Särkkä, S. and Vehtari, A. (2014). MCMC Diagnostics Toolbox for Matlab.

[26] Schellenberger, J., Que, R., Fleming, R. M. T., Thiele, I., Orth, J. D., Feist, A. M., Zielinski, D. C., Bordbar, A., Lewis, N. E., Rahmanian, S., Kang, J., Hyduke, D. R., and Palsson, B. Ø. (2011). Quantitative prediction of cellular metabolism with constraint-based models: the COBRA Toolbox v2.0. *Nat. Protoc.*, **6**(9), 1290–1307.

[27] Sonnevend, G. (1990). Applications of analytic centers for the numerical solution of semi-infinite, convex programs arising in control theory. In *System Modelling and Optimization (Leipzig, 1989)*, volume 143 of *Lecture Notes in Control and Inform. Sci.*, pages 413–422. Springer, Berlin, Germany.

[28] Thiele, I., Vo, T. D., Price, N. D., and Palsson, B. Ø. (2005). Expanded metabolic reconstruction of Helicobacter pylori (iIT341 GSM/GPR): an in silico genome-scale characterization of single- and double-deletion mutants. *J. Bacteriol.*, **187**(16), 5818–5830.

[29] Thiele, I., Swainston, N., Fleming, R. M. T., Hoppe, A., Sahoo, S., Aurich, M. K., Haraldsdóttir, H., Mo, M. L., Rolfsson, O., Stobbe, M. D., Thorleifsson, S. G., Agren, R., Bölling, C., Bordel, S., Chavali, A. K., Dobson, P., Dunn, W. B., Endler, L., Hala, D., Hucka, M., Hull, D., Jameson, D., Jamshidi, N., Jonsson, J. J., Juty, N., Keating, S., Nookaew, I., Le Novère, N., Malys, N., Mazein, A., Papin, J. A., Price, N. D., Selkov Sr, E., Sigurdsson, M. I., Simeonidis, E., Sonnenschein, N., Smallbone, K., Sorokin, A., van Beek, J. H. G. M., Weichart, D., Goryanin, I., Nielsen, J., Westerhoff, H. V., Kell, D. B., Mendes, P., and Palsson, B. Ø. (2013). A community-driven global reconstruction of human metabolism. *Nat. Biotechnol.*, **31**(5), 419–425.

[30] Thomas, A., Rahmanian, S., Bordbar, A., Palsson, B. Ø., and Jamshidi, N. (2014). Network reconstruction of platelet metabolism identifies metabolic signature for aspirin resistance. *Sci. Rep.*, **4**, 3925.

[31] Zhang, Y., Thiele, I., Weekes, D., Li, Z., Jaroszewski, L., Ginalski, K., Deacon, A. M., Wooley, J., Lesley, S. a., Wilson, I. a., Palsson, B., Osterman, A., and Godzik, A. (2009). Three-dimensional structural view of the central metabolic network of Thermotoga maritima. *Science*, **325**(5947), 1544–1549.
